# Prediction of recurrence free survival for esophageal cancer patients using a protein signature based risk model

**DOI:** 10.18632/oncotarget.10656

**Published:** 2022-09-14

**Authors:** Raghibul Hasan, Gunjan Srivastava, Akram Alyass, Rinu Sharma, Anoop Saraya, Tushar K. Chattopadhyay, Siddartha DattaGupta, Paul G. Walfish, Shyam S. Chauhan, Ranju Ralhan

**Affiliations:** ^1^Department of Biochemistry, All India Institute of Medical Sciences, Ansari Nagar, New Delhi, India; ^2^Alex and Simona Shnaider Research Laboratory in Molecular Oncology, Department of Pathology and Laboratory Medicine, Mount Sinai Hospital, Toronto, Ontario, Canada; ^3^Department of Clinical Epidemiology and Biostatistics, McMaster University, Hamilton, Ontario, Canada; ^4^University School of Biotechnology, Guru Gobind Singh Indraprastha Univesity, Dwarka, New Delhi, India; ^5^Department of Gastroenterology, All India Institute of Medical Sciences, Ansari Nagar, New Delhi, India; ^6^Department of Gastrointestinal Surgery, All India Institute of Medical Sciences, Ansari Nagar, New Delhi, India; ^7^Department of Pathology, All India Institute of Medical Sciences, Ansari Nagar, New Delhi, India; ^8^Department of Medicine, Endocrine Division, Mount Sinai Hospital and University of Toronto, Toronto, Ontario, Canada; ^9^Department of Pathology and Laboratory Medicine, Mount Sinai Hospital, Toronto, Ontario, Canada; ^10^Joseph and Mildred Sonshine Family Centre for Head and Neck Diseases, Department of Otolaryngology – Head and Neck Surgery, Mount Sinai Hospital, Toronto, Ontario, Canada; ^11^Department of Otolaryngology – Head and Neck Surgery, University of Toronto, Toronto, Ontario, Canada

**Keywords:** esophageal cancer, wnt proteins, dishevelled, molecular markers, prognosis

## Abstract

Background: Biomarkers to predict the risk of disease recurrence in Esophageal squamous cell carcinoma (ESCC) patients are urgently needed to improve treatment. We developed proteins expression-based risk model to predict recurrence free survival for ESCC patients.

Methods: Alterations in Wnt pathway components expression and subcellular localization were analyzed by immunohistochemistry in 80 ESCCs, 61 esophageal dysplastic and 47 normal tissues; correlated with clinicopathological parameters and clinical outcome over 86 months by survival analysis. Significant prognostic factors were identified by multivariable Cox regression analysis.

Results: Biomarker signature score based on cytoplasmic β-catenin, nuclear c-Myc, nuclear DVL and membrane α-catenin was associated with recurrence free survival [Hazard ratio = 1.11 (95% CI = 1.05, 1.17), *p* < 0.001, C-index = 0.68] and added significant prognostic value over clinical parameters (*p* < 0.001). The inclusion of Slug further improved prognostic utility (*p* < 0.001, C-index = 0.71). Biomarker Signature Score_slug_ improved risk classification abilities for clinical outcomes at 3 years, accurately predicting recurrence in 79% patients in 1 year and 97% in 3 years in high risk group; 73% patients within low risk group did not have recurrence in 1 year, with AUC of 0.76.

Conclusions: Our comprehensive risk model predictive for recurrence allowed us to determine the robustness of our biomarker panel in stratification of ESCC patients at high or low risk of disease recurrence; high risk patients are stratified for more rigorous personalized treatment while the low risk patients may be spared from harmful side effects of toxic therapy.

## INTRODUCTION

Esophageal cancer (EC), one of the most life threatening malignancies of the upper aero-digestive tract, is the 8th most common cancer in the world (GLOBOCAN 2013) with 456,000 new cases in 2012 (3.2% of the total). It is the sixth most common cause of death from cancer with an estimated 400,000 deaths (4.9% of the total), with the developing countries bearing nearly 80% of the total burden of this cancer. EC demonstrates wide variation in geographical incidence owing to differences in etiological factors. There are two main forms of esophageal cancer, esophageal squamous cell carcinoma (ESCC) and esophageal adenocarcinoma (EA). Both are characterized by distinct etiological and pathological characteristics. Despite advances in multimodality therapy, because of insidious symptomatology, late stage of diagnosis and poor efficacy of treatment, the prognosis for patients with ESCC still remains poor, with an average 5-year survival of < 10% globally [1, 2]. So far, very few genetic alterations have been directly linked to esophageal tumorigenesis.

Wnt signalling regulates cell growth, motility and differentiation and is an important player in cancer development [3–5]. β-catenin is an integral component in Wnt signalling pathway. β-catenin is stabilized and accumulates in the cytosol which, in association with T-cell factor/lymphoid enhancer binding factor (TCF/Lef), translocate to the nucleus and induces transcription of Wnt target genes. In the nucleus, Dvl has been found to interact with c-Jun and β-catenin, followed by formation of stable β-catenin/TCF complex and transcriptional activation of Wnt target genes [3, 6]. In cancer cells, mutations or abnormal promoter methylation of genes encoding components of the Wnt signalling system, such as adenomatous polyposis coli (APC), Axin, or β-catenin, lead to abnormal activation of Wnt-induced transcription and overexpression of genes involved in tumorigenesis. Recent whole genome sequencing studies from the International Cancer Genome Consortium Research reported somatic aberrations in the Wnt, cell cycle and Notch pathways as the key players in esophageal tumorigenesis [1, 2]. In a parallel study we had hypothesized that alterations in expression of Wnt proteins in ESCC are likely to be associated with disease outcome and may be used to predict recurrence free survival in these patients. To address this hypothesis we analyzed alterations in expression and subcellular localization of the key components of Wnt pathway including β-catenin, α-catenin, dishevelled (DVL), c-Myc and compared with E-cadherin, in a series of ESCCs, esophageal dysplasia and histologically normal esophageal tissues by immunohistochemistry. The alterations in expression of these proteins were correlated with clinico-pathological parameters of ESCC patients and follow-up over a period of up to 86 months for assessment of their prognostic relevance. Based on our findings, we developed a protein expression-based risk prediction model for recurrence free survival of ESCC patients, as a step forward towards establishing their clinical applicability that is likely to have implications for personalized therapy.

## RESULTS

### Patients and treatment

Table 1 summarizes the demographics and clinicopathological parameters of 80 ESCC cases selected for our analysis. The median age of the study population is 54.5 years (range 23–80 years). Lymph node positivity was observed in 56 of 80 (70%) patients. All patients received surgery as the primary treatment. Post-surgery treatments were performed according to the NCCN guidelines.

**Table 1:** Clinicopathological characteristics of ESCC patients’ cohort

Total ESCC cases	80
**Age at diagnosis (years)**	
Range	23–80
Median ± S.D.	54.5 ± 12.3
**Gender**	
Male	52 (65%)
Female	28 (35%)
**AJCC pTNM Stage**	
I and II	8 (10%)
III and IV	72 (90%)
**Nodal status**	
Negative	24 (30%)
Positive	56 (70%)
**Histology grade**	
WDSCC	25 (31.25%)
MDSCC	42 (52.5%)
PDSCC	13 (16.25%)
**Treatment**	
Surgery	28 (35%)
Surgery + Radiation therapy	46 (57.5%)
Surgery + Chemotherapy	2 (2.5%)
Surgery + Radiation and Chemotherapy	4 (5%)

### Evaluation of biomarkers expressions in esophageal tissues by immunohistochemistry

**Figure 1:**
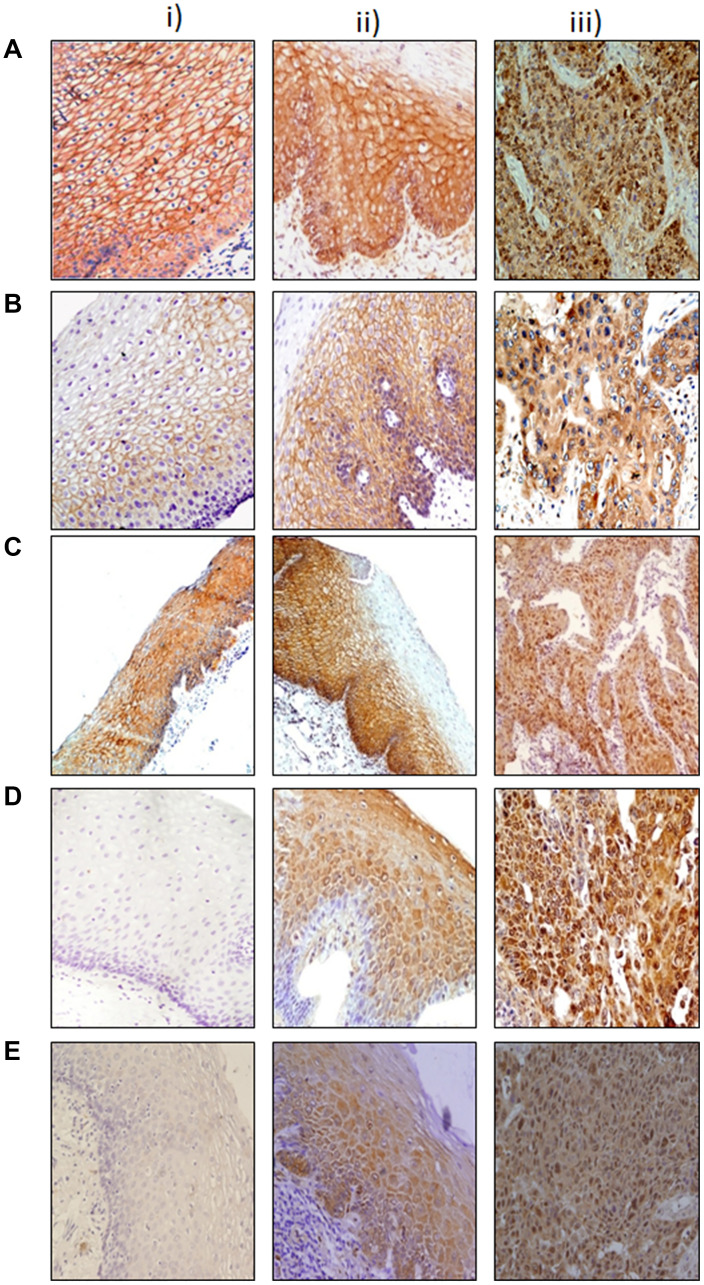
Immunohistochemical analysis of Wnt protein in esophageal tissues. Paraffin-embedded sections of histological normal mucosa, dysplasia, and ESCC were stained with: (**A**) β-catenin i) histologically normal tissue showing strong membranous immunoreactivity; ii) dysplasia showing strong membranous and cytoplasmic immunoreactivity: iii) ESCC showing nuclear immunoreactivity. (**B**) E-cadherin i) histologically normal tissue showing intense membranous staining; ii) dysplasia tissue showing membrane and cytoplasmic immunostaining: iii) ESCC tissue showing cytoplasmic staining. (**C**) α-catenin i) histologically normal tissue showing intense membranous immunoreactivity; ii) dysplasia tissue showing strong membranous immunoreactivity; iii) ESCC tissue showing membrane loss and cytoplasmic immunoreactivity. (**D**) Dishevelled (DVL) (i) histologically normal tissue showing no immunoreactivity; ii) dysplasia tissue showing cytoplasmic/nuclear immunoreactivity; iii) ESCC tissue showing intense nuclear/cytoplasmic immunoreactivity. (**E**) i) c-Myc histologically normal tissue showing no immunoreactivity; ii) dysplasia tissue showing cytoplasmic immunoreactivity; iii) ESCC tissue showing intense nuclear immunoreactivity (original magnification A–E ×200).

Representative photomicrographs depicting alterations in membrane, cytoplasmic and nuclear expressions of β-catenin, E-cadherin, α-catenin, c-Myc, and DVL in esophageal dysplasia and ESCC as compared to the normal esophageal tissues are shown in Figure 1. The panels A (i–iii) show significant loss of membranous β-catenin in dysplasia and ESCC with concomitant increase in cytoplasmic levels in comparison with normal esophageal tissues. Similar increase in cytoplasmic levels of E-cadherin, α-catenin, c-Myc, and DVL was observed in esophageal dysplasia and ESCC as compared to the normal esophageal tissues, while nuclear expression of c-Myc and DVL was also observed in these tissues as shown in panels B (i–iii) to E (i–iii) respectively. Representative photomicrographs of negative and positive controls used for immunostaining for each protein are given in (Supplementary Figure 1). The subcellular compartmental expressions of β-catenin, E-cadherin, α-catenin, c-Myc, and DVL are summarized in Table 2. Membrane expression levels of β-catenin, E-cadherin, and α-catenin were significantly reduced in ESCCs as compared to dysplasia (*p* < 0.001), and in dysplasia compared to normal tissues except for membrane β-catenin (*p* = 0.17, *p* < 0.001, *p* = 0.001 respectively). In comparison with normal tissues, dysplasia showed increased expression levels of cytoplasmic β-catenin, E-cadherin, α-catenin (*p* < 0.001), c-Myc, and DVL (*p* = 0.02, *p* = 0.009 respectively), and in ESCC compared to dysplasia tissues (*p* < 0.001) with the exception of cytoplasmic E-cadherin. Nuclear expression of both c-Myc, and DVL were found to increase significantly in ESCC compared to dysplasia (*p* < 0.001); nuclear c-Myc expression was also increased in dysplasia compared to normal tissues (*p* = 0.007).

**Table 2:** Immunohistochemical analysis in esophageal tissues

Tissues	*n*	Membrane expression mean (SD)	*p*^†^	Cytoplasmic expression mean (SD)	*p*^†^	Nuclear expression mean (SD)	*p*
**β-catenin**							
Normal	47	5.26 (1.82)	0.17	0.00 (0.00)	< 0.001	ND^*^	0.08
Dysplasia	61	4.18 (2.74)	< 0.001	0.98 (1.86)	< 0.001	0.20 (0.77)	0.006
ESCC	80	0.84 (1.91)	< 0.001	2.56 (2.67)	0.0003	0.71 (1.79)	0.09
**E-cadherin**							
Normal	47	5.13 (2.50)	< 0.001	0.00 (0.00)	< 0.001	ND^*^	
Dysplasia	61	3.07 (2.63)	< 0.001	1.13 (2.00)	0.001	ND^*^	
ESCC	80	0.79 (1.78)	< 0.001	0.94 (1.96)	0.49	ND^*^	
**α-Catenin**							
Normal	47	5.64 (1.65)	0.001	0.74 (1.67)	< 0.001	ND^*^	
Dysplasia	61	4.28 (2.39)	< 0.001	2.21 (2.38)	< 0.001	ND^*^	
ESCC	80	1.29 (2.38)	< 0.001	4.28 (2.34)	< 0.001	ND^*^	
**c-Myc**							
Normal	47	ND^*^		0.4 (1.01)	0.02	0.30 (0.83)	0.007
Dysplasia	61	ND^*^		1.36 (2.15)	< 0.001	1.31 (2.08)	< 0.001
ESCC	80	ND^*^		4.00 (2.77)	< 0.001	3.33 (2.78)	< 0.001
**DVL**							
Normal	47	ND^*^		1.11 (1.96)	0.009	0.00 (0.00)	---
Dysplasia	61	ND^*^		2.34 (2.71)	< 0.001	0.00 (0.00)	< 0.001
ESCC	80	ND^*^		4.96 (2.66)	< 0.001	1.36 (2.53)	< 0.001

Abbreviation: ^*^ND: Not detectable. ^†^normal vs. dysplasia/normal vs. cancer/dysplasia vs. cancer.

### Prognostic value and clinical relevance of biomarkers

**Figure 2:**
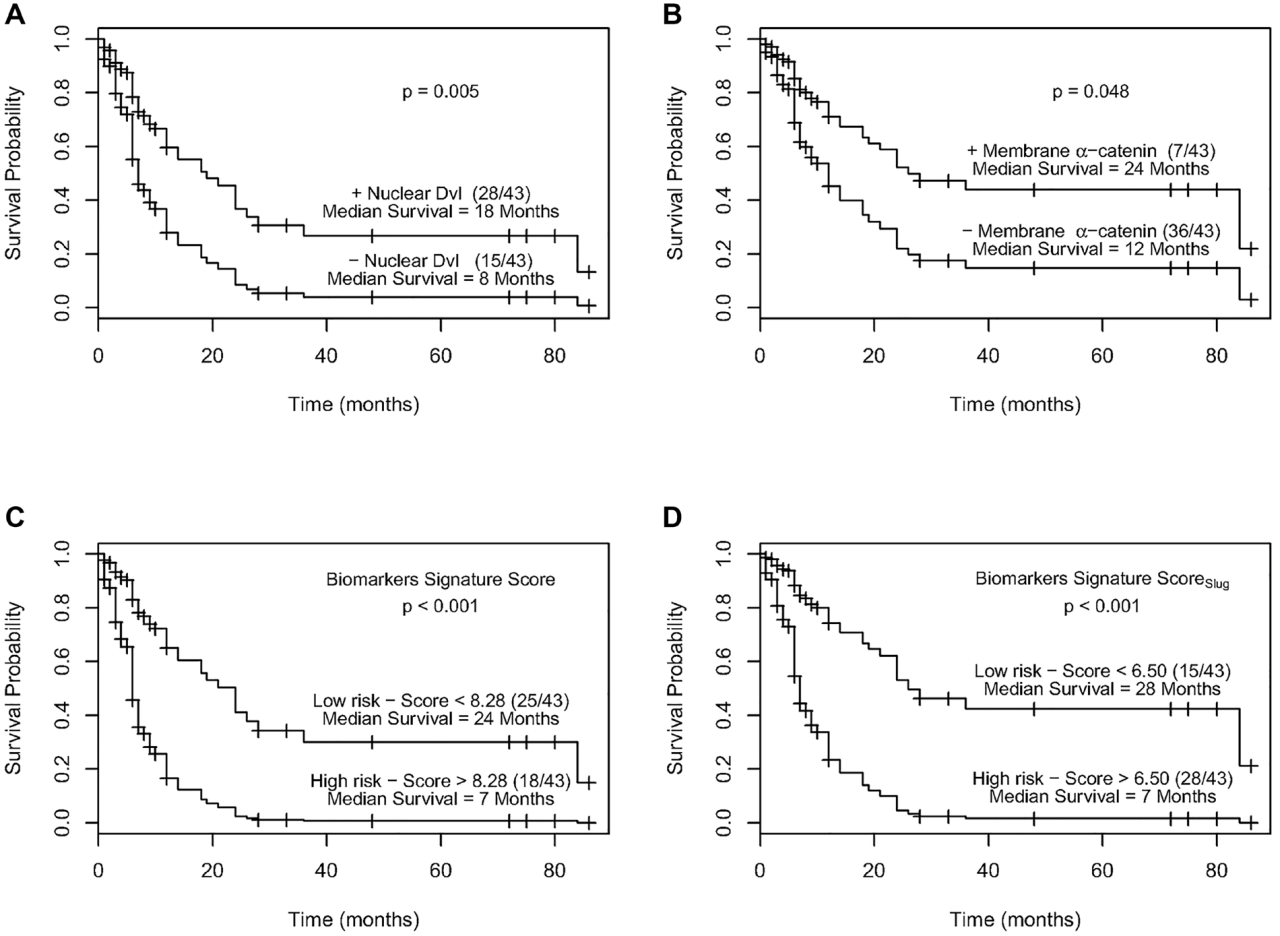
(**A)** Kaplan–Meier estimation of cumulative proportion of disease-free survival. Median time for disease-free survival (DFS; no recurrence/metastasis) in ESCC patients showing nuclear immunopositivity of Dvl was 18 months as compared to 8 months for the patients who didn’t show nuclear Dvl immunostaining (*p* = 0.005). (**B**) Kaplan–Meier estimation of cumulative proportion of disease-free survival. Median time for disease-free survival (DFS; no recurrence/metastasis) in ESCC patients showing immunostaining of membrane α-catenin was 24 months as compared to 12 months for the patients who didn’t show membrane α-catenin immunopositivity (*p* = 0.048). (**C**) Survival curves of high and low recurrence risk groups in ESCC using biomarker signature score. The median survival time for patients with a biomarker signature score less than 8.28 (low risk group) was 24 months as compared to 7 months for patients with a score greater than 8.28 (high risk group) (*p* < 0.001). (**D**) Survival curves of high and low recurrence risk groups in ESCC using biomarker signature score_Slug_. The median survival time for patients with a biomarker signature score_Slug_ less than 6.50 (low risk group) was 28 months as compared to 7 months for patients with a score greater than 6.50 (high risk group) (*p* < 0.001).

To identify the best panel of prognostic biomarkers, various Cox regression models were fitted based on nuclear c-Myc, cytoplasmic DVL, nuclear DVL, membrane β-catenin, cytoplasmic β-catenin, and membrane α-catenin (Supplementary Table 1). A panel of markers comprising of cytoplasmic β-catenin, nuclear c-Myc, nuclear DVL and membrane α-catenin achieved the best model fit to assess disease prognosis (*p* = 0.009, c-statistic = 0.68, Table 3). Cytoplasmic β-catenin, and nuclear DVL were associated with poor disease prognosis in univariate analyses (*p* = 0.01, *p* = 0.02 respectively, Table 3), and in multivariate analyses adjusted for tumor stage, nodal status, histology grade and treatment (*p* = 0.007, *p* = 0.04 respectively). Membrane α-catenin was associated with good prognosis adjusted for clinical parameters as well, while nuclear c-Myc was marginally significant (Table 3). Furthermore, Kaplan Meier analyses showed reduced nuclear DVL and membrane α-catenin were associated with shorter recurrence free survival (*p* = 0.005 and 0.048 respectively) (Figure 2A and 2B).

**Table 3:** Univariate and multivariable cox analyses

Predictors^*^	Univariate analyses		Multivariable analyses	
	HR [95% CI]	*p*	HR [95% CI]	*p*
Cytoplasmic β-catenin	1.16 [1.03, 1.30]	0.01	1.19 [1.09, 1.35]	0.007
Membrane α-catenin	0.87 [0.74, 1.01]	0.07	0.84 [0.71, 0.99]	0.04
Nuclear DVL	1.13 [1.02, 1.26]	0.02	1.11 [1.01, 1.24]	0.04
Nuclear c-Myc	1.08 [0.96, 1.21]	0.20	1.12 [0.99, 1.27]	0.07
**Biomarker Signature Score**	1.11 [1.05, 1.17]	< 0.001	1.11 [1.05, 1.17]	< 0.001
Nuclear Slug	1.21 [1.08, 1.37]	0.002	1.20 [1.06, 1.37]	0.009
**Biomarker Signature Score_slug_**	1.11 [1.05, 1.16]	< 0.001	1.10 [1.05, 1.16]	< 0.001
Tumor stage	2.85 [0.69, 11.81]	0.15		
Nodal Status	1.77 [0.89, 3.55]	0.11		
Histological grade	1.07 [0.55, 2.08]	0.84		
Radiation therapy	0.73 [0.39, 1.39]	0.34		
Chemotherapy	0.90 [0.53, 1.53]	0.69		
**Predictors**	**c-statistic**		**Model LRT p/(AIC)**	
**Biomarker Signature Score**				
cytoplasmic β-catenin + nuclear c-MyC + nuclear DVL + membrane α-catenin	0.68		0.009/(291.52)	
**Biomarker Signature Score_slug_**				
cytoplasmic β-catenin + nuclear c-MyC + nuclear DVL + nuclear Slug	0.71		0.005/(290.13)	

^*^All multivariable Cox regression models were adjusted for tumor stage, nodal status, histological grade, and treatment. Internal validation based on 9999 bootstrap samples yielded similar results.

### Biomarkers signature score

A signature score based on biomarkers expression levels with regression estimates as weights (Biomarker Score = 1.03 × cytoplasmic β-catenin + 0.73 × nuclear c-Myc + nuclear 1.07 × DVL – 1.22 × α-catenin membrane) was strongly associated with prognosis of ESCC recurrence/death [HR = 1.11 (95% CI = 1.05, 1.17), *p* < 0.001, c-statistic = 0.68, Table 3]. This signature score was associated with prognosis adjusted for clinicopathological parameters and treatment as well (*p* < 0.001). Bootstrap analyses based on 9999 sample sets gave similar Cox regression HR (95% CI) thereby validating these findings (Supplementary Table 2; Supplementary Figure 2A).

Recursive partitioning (RP) analyses showed a significant difference in survival curves of ESCC patients based on a cut-off value of 8.28 (Figure 2C). Participants with a Biomarker signature score greater than 8.28 had a 1-year survival rate of 17% (95% CI = 6%, 47%), none of the patients survived in 3 years and had a median survival time of 7 months (95% CI = 4, 12) (Figure 2C). This is compared to a 1year survival rate of 65% (95% CI = 51%, 81%), a 3 years survival rate of 31% (95% CI = 18%, 54%) and a median survival time of 24 months (95% lower bound = 14 months) in patients stratified to the low risk group (score less than 8.28).

The clinical relevance of these biomarkers’ signature score was assessed in correctly identifying subjects at high and low risk of ESCC recurrence/death within 1 year and 3 years post-surgery (Table 4). Using a cut-off value of 8.28, 84% and 100% patients who did not show recurrence/death were correctly stratified into the low risk group within 1 year and 3 years respectively. In the high risk group 79% cases had cancer recurrence/death events within 1 year, where as in 3 years, all the patients developed recurrence or died. The AUC of this risk stratification was 0. 67 at 1 year and 0.73 at 3 years. Internal validation based on 9999 bootstrap samples confirmed the clinical relevance of this Biomarker signature score (Supplementary Table 3). In comparison, risk stratification based on clinicopathological parameters (tumor stage, histology grade, and nodal status) achieved an AUC of 0.58 in 1year in a similar analysis (data not shown).

**Table 4:** Clinical relevance of biomarker signature scores

Clinical value	Biomarker signature score	Biomarker signature score_slug_
**1 Year**		
	High vs. Low Risk Groups	High vs. Low Risk Groups
Sensitivity	0.50	0.77
Specificity	0.84	0.76
PPV	0.79	0.79
NPV	0.58	0.73
AUC	0.67	0.76
**3 Years**		
Sensitivity	0.45	0.67
Specificity	1.00	0.85
PPV	1.00	0.97
NPV	0.23	0.30
AUC	0.73	0.76

Biomarker Signature Score refers to cytoplasmic β-catenin, nuclear c-Myc, nuclear DVL and membrane α-catenin signature score (cut-off value = 8.28). Biomarker Signature Score_slug_ refers Score with nuclear Slug instead of membrane α-catenin (cut-off value = 6.50). Internal validation based on 9999 bootstrap samples yielded identical values for Sensitivity, Specificity, PPV, NPV and AUC.

### Biomarkers signature score_slug_

Recently, using the same patient cohort we reported alterations in Slug expression occur in early stages of development of ESCC and are sustained during disease progression [7]. Slug was found to be a predictor of poor disease prognosis to identify ESCC patients that are likely to show recurrence of the disease. Here in we investigated if inclusion of Slug in our current biomarker panel of Wnt proteins would improve their performance for risk prediction of ESCC patients. Nuclear Slug was found to be associated with disease prognosis in univariate analyses [HR = 1.21 (95% CI = 1.08, 1.37), *p* = 0.002], and when adjusted for tumor stage, histology grade, nodal status, and treatment [HR = 1.20 (95% CI = 1.06, 1.37), *p* = 0.009, Table 3]. A panel consisting of cytoplasmic β-catenin, nuclear c-Myc, nuclear DVL and nuclear Slug achieved the best model fit (*p* = 0.005, c-statistic= 0.71, Table 3). A signature score based on these biomarkers using their regression estimates as weights (Biomarker Signature Score_slug_ = 0.72× cytoplasmic β-catenin + 0.72× nuclear c-Myc + 0.74× nuclear DVL – 1.46× nuclear Slug) was associated with disease prognosis [HR = 1.11 (95% CI = 1.05, 1.16), *p* < 0.001, c-statistic = 0.71, Table 3]. These results were internally validated based on 9999 bootstrap samples (Supplementary Table 2; Supplementary Figure 2B).

Recursive partitioning (RP) analyses on Biomarker Signature Score_slug_ identified two nodes. ESCC patients in the high risk group (with a score greater than 6.50) were found to have a 1-year survival rate of 22% (95% CI = 11%, 45%), a 3 year survival rate of 4% (95% CI = 1.00%, 26%), and a median survival time of 7 months (95% CI = 6, 12) (Figure 2D). Patients in the low risk group had a 1year survival rate of 76% (95% CI = 62%, 94%), a 3 year survival rate of 39%, (95% CI = 23% 68%), and a median survival time of 28 months.

The clinical relevance of the Biomarker Signature Score_slug_ using a cut-off value of 6.50 for stratifying high and low risk groups had an improved AUC 0f 0.76 for 1 and 3 years post-surgery (Table 4). Seventy nine percent of subjects in the high risk group developed disease recurrence/death within one year post surgery, while 73% of the low risk group did not. With 3 years follow up, the number of recurrence/death cases increased, and 97% of subjects within the high risk group had recurrence. Internal validation based on 9999 bootstrap samples confirmed the clinical relevance of the Biomarker Signature Score_slug_ (Supplementary Table 3). The predictive accuracy of Biomarker Signature Score_slug_ within 1 year outperformed the accuracy of the clinical score, (*p* = 0.01).

## DISCUSSION

Currently, there are limited clinical approaches for early diagnosis and treatment of ESCC, resulting in a five-year survival rate of 10% for these patients [2]. The full repertoire of molecular events leading to pathogenesis of ESCC remains unclear. Recently Song et al., [1, 2] based on a comprehensive genomic analysis of 158 ESCC cases reported that ESCC and HNSCC pathogenesis may share many common characteristics including activation of MAPK, JAK-STAT, Wnt, NOTCH pathways and CNA profiles of many genes in cell cycle. Notably, altered genes in the Wnt pathway were detected in 86.4% of ESCCs including mutations in CTNNB1, SFRP4, DVL and Yap1. Epigenetic inactivation has been reported for SFRP2 in ESCC [8]. Using next-generation sequencing technology for extensive mutation analysis and bead-array technology for genome-wide DNA methylation analysis on the same tumor samples [9], ESCCs were recently characterized and WNT pathway was reported to be a key altered pathway in ESCC, activated potentially by aberrant methylation of its negative regulators, such as SFRP1, SFRP2, SFRP4, SFRP5, SOX17, and WIF1 (33%). However, the clinical relevance of these recent findings remains to be established. Few studies have examined the clinical relevance of biomarkers in ESCC, but a major limitation of most of these reports is evaluation of single proteins as biomarkers [10–12]. Our study is important because: (i) it is based on changes in expression levels of the biomarker proteins in different subcellular compartments and is not limited to alterations in the overall protein expression levels; (ii) investigates the comprehensive clinical relevance of subcellular alterations in expression of multiple key components of Wnt pathway in the same ESCC patients’ cohort; (iii) correlates these findings with disease outcome and (iv) develops a Biomarker risk score for defining the risk of recurrence of ESCCs.

**Figure 3:**
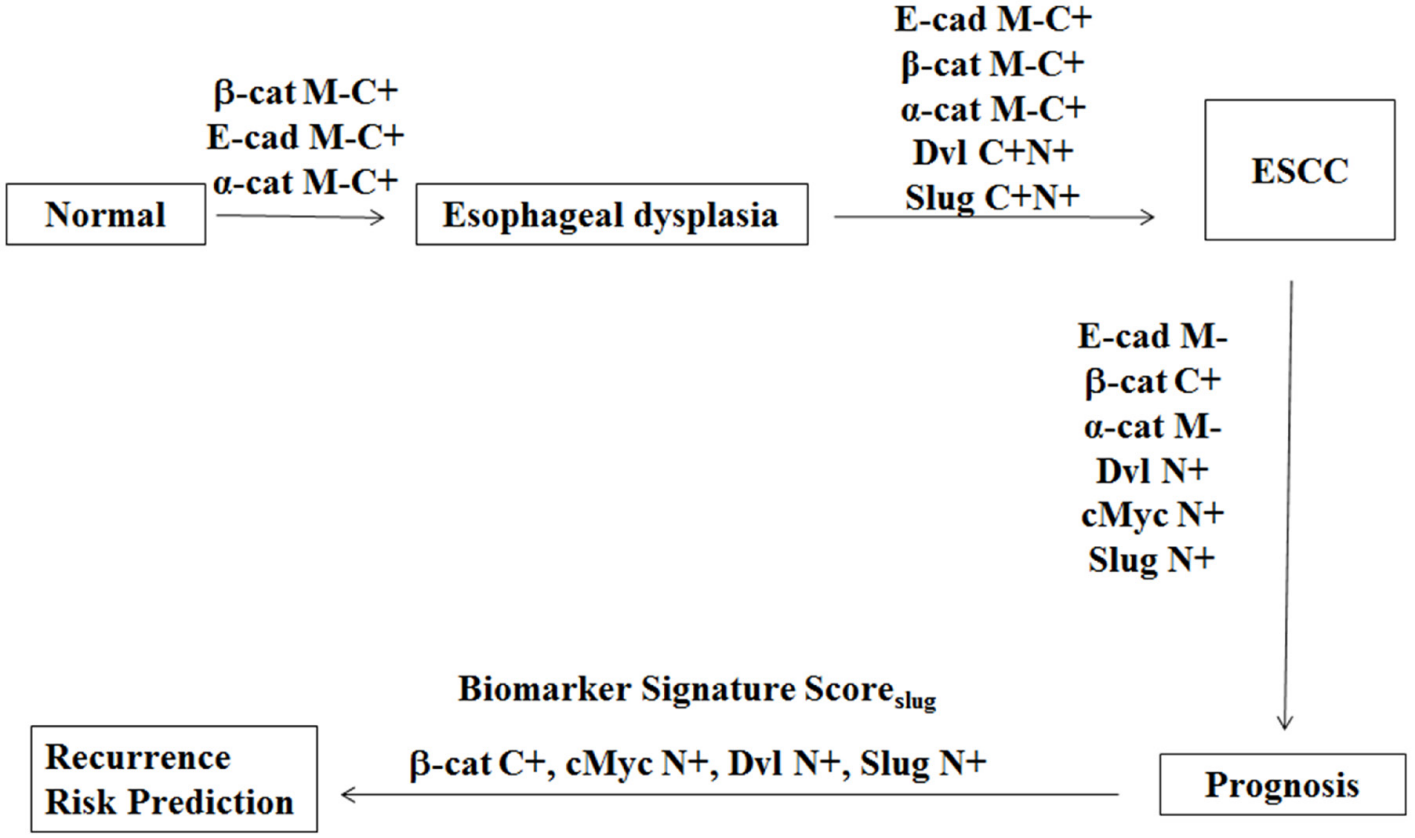
Schematic diagram depicting alterations in proteins expression in multistep esophageal tumorigenesis. Esophageal dysplasia show significant loss of membrane and accumulation in cytoplasmic E-cadherin, β-catenin and α-catenin in comparison with the normal esophageal tissues. ESCC show marked increase in cytoplasmic and nuclear DVL and Slug as compared to dysplasia. The prognosis of ESCC is associated with loss of membranous E-cadherin and α-catenin and accumulation of cytoplasmic β-catenin, as well as nuclear accumulation of DVL, cMyc and Slug. A Biomarker Signature Score_Slug_ based on cytoplasmic β-catenin and nuclear cMyc, DVL and Slug has been used to develop a risk classifier for prediction of recurrence free survival for ESCC patients. E-cad M-, E cadherin membrane loss; Slug C+, Slug cytoplasmic expression; Slug N+, Slug nuclear expression; α-cat M-, alpha catenin membrane loss; α-cat C+, alpha catenin cytoplasmic expression; β-cat M-, beta catenin membrane loss; β-cat C+, beta catenin cytoplasmic expression; cMyc N+, cMyc nuclear expression.

A schematic diagram depicting alterations in subcellular compartmental localization of the proteins investigated in multistep esophageal tumorigenesis in our study is shown in Figure 3. Esophageal dysplasia showed significant loss of membrane and accumulation in cytoplasmic E-cadherin, β-catenin and α-catenin in comparison with the normal esophageal tissues that is sustained in ESCC revealing the potential of these proteins as biomarkers for distinguishing dysplasia from normal esophageal tissues. To our knowledge this is the first comprehensive study demonstrating cytoplasmic accumulation of β-catenin, E-cadherin, α-catenin and cytoplasmic DVL as early as in dysplasia during the multi-step process of esophageal tumorigenesis suggesting these alterations in Wnt pathway components occur in early stages during the development of ESCC. The loss of E-cadherin, β-catenin and α-catenin from the membrane of epithelial cells in dysplasia suggests a disruption in the epithelial tissue architecture that might play an important role in epithelial mesenchymal transition (EMT) and tumor invasion. β-Catenin is the key mediator of cellular responses to Wnt signalling, and its cytoplasmic/nuclear partitioning is tightly controlled in cancer including ESCC [13].

Another important finding of our study is the significant increase in cytoplasmic and nuclear Dvl in ESCC as compared to dysplastic esophageal tissues underscoring its relevance in progression of the disease. In support of our findings Dvl has been implicated in oncogenesis and is overexpressed in prostate cancer, non-small cell lung cancer, mesothelioma and colon cancer; its upregulation has been implicated to be correlated with activation of Wnt/β-catenin signaling and an increased risk of malignant transformation [14–17]. Notably, nuclear localization of DVL has been reported in colon cancer as well. Wang et al., [18] reported that FOXKs promote Wnt/β-catenin signaling by translocating DVL into the nucleus. FOXK proteins activate Wnt/β-catenin signaling by promoting DVL nuclear translocation, which requires the Wnt signalling-induced DVL phosphorylation. Further the authors showed that FOXK protein expression is significantly increased in human colon cancers and correlates with DVL nuclear localization. Using a transgenic mouse model, for conditional expression of Foxk2, induced intestinal hyper-proliferation, nuclear translocation of DVL and up-regulation of Wnt/β-catenin signaling in the intestine crypts. These authors proposed a potential oncogenic function of FOXK proteins via their abilities to translocate DVL into the nucleus and activate the Wnt/β-catenin signalling pathway. Our study showed cytoplasmic/nuclear accumulation of c-Myc is increased in ESCC as compared to dysplasia and correlates with poor prognosis and in support of our findings c-Myc overexpression in prostate and laryngeal cancer has been reported to predict biochemical recurrence [19, 20].

Our study also assumes importance in view of the vital role played by the Wnt signaling pathway components in chemo-radiation resistance of ESCC and their emerging utility as novel therapeutic targets. The expression of paired-like homeodomain transcription factor 2 (PITX2), a downstream effector of Wnt/β-catenin signaling, was observed more frequently in chemoradiotherapy (CRT) resistant ESCC patients than that in CRT effective group (*p* < 0.05) and was associated with poor disease-specific survival (*p* < 0.05). Thus, PITX2 expression may be a useful tool for predicting CRT resistance and serves as an independent molecular marker for poor prognosis of ESCC patients treated with definite CRT [21]. Recently, NHE9 has been shown to induce CRT resistance in ESCC by upregulating the Src/Akt/β-catenin pathway and Bcl-2 expression suggesting its potential as an effective predictor of CRT response and may be useful in the development of targeted therapies for CRT-resistant ESCC [22]. The role of esophageal cancer stem cells in regulating Wnt signaling pathway and chemo-radiation resistance and implications for designing novel targeted therapies has been recently reviewed [23].

Importantly, we demonstrated that a panel of 4 biomarkers, cytoplasmic β-catenin, nuclear c-Myc, nuclear DVL and membrane α-catenin, constituted the prognostic molecular signature for ESCC patients. Our panel of biomarkers predicted disease recurrence more effectively as compared to individual biomarkers analyzed in this study and demonstrated the strong predictive power of this panel of biomarkers for ESCC patients. We have demonstrated the clinical usefulness of this promising panel of biomarkers by their ability to add unique prognostic information to the clinical predictors – histological grade, nodal status tumor stage and treatment.

Slug has recently been demonstrated to be a target gene in the signalling cascade TGF-β-PI3K/Akt-GSK3β-Snail-Slug-CD147, involved in epithelial mesenchymal transition and is implicated in hepatocarcinogenesis and hepatocellular carcinoma metastasis [24]. TGF-β has been reported to play important role in chronic inflammation and esophageal tumorigenesis [25–27]. These reports and our recent study on Slug expression in ESCC [28] provided the rationale to investigate the potential of Slug as a predictive marker in combination with our current panel of biomarkers. Notably, the risk stratification based on inclusion of Slug in the Biochemical signature score (AUC 0.76) was more accurate than using the biomarkers signature score with α-catenin (AUC 0.67). Biomarker Signature Score_slug_ accurately predicted recurrence in 79% patients in 1 year and 97% in 3 years in the high risk group, while 73% patients within the low risk group did not have recurrence in 1 year, suggesting its applicability for risk stratification.

However, our study is not devoid of limitations. First, the end point of this study was disease recurrence. Although this is a surrogate end point for clinical progression, not all patients with recurrence will progress to distant metastases and/ or cancer-related death. Unfortunately, the natural history of esophageal cancer limits the availability of more definitive end points. Despite these limitations, we were able to demonstrate a highly statistically significant relationship between these biomarkers of interest, and also showed better accuracy of the biomarkers in identifying ESCC patients at higher risk of recurrence of the disease. Further, the clinical implementation of our panel of Biomarker signature score_slug_ based test uses the technique of immunohistochemical analysis that is routinely performed in most pathological laboratories and thus easy to translate from bench to clinic. Another major advantage of our proteins based test panel is its cost effectiveness which is generally less than the cost of gene signature based tests.

The internal validations demonstrate the stability of our Biomarker signature score_slug_ utility in clinical settings. These findings set the stage for independent multicentric prospective studies to assess if this risk classifier could help to predict recurrence free survival that can be used to guide clinical management of ESCC in future.

## MATERIALS AND METHODS

### Study design

This study is part of a single institution project initiated in 2005, aimed to identify novel biomarkers predicting disease progression in ESCC patients. The study is conducted according to the Reporting of tumor MARKer Studies (REMARK) guidelines and a prospectively written research, pathologic evaluation, and statistical analysis plan. This study was approved by the All India Institute of Medical Sciences (AIIMS) Research Ethics Board, New Delhi, India prior to its commencement. Written informed consent was obtained for the acquisition and use of patient tissue samples and anonymized clinical data. Tissue specimens were obtained by diagnostic or therapeutic procedures from 61 patients with clinically defined esophageal dysplasia attending the Outpatient Clinic of the Departments of Surgical Disciplines and Gastrointestinal Surgery, AIIMS. We selected among the 150 ESCC patients undergoing curative cancer surgery during the period 2005 – 2010, 80 consecutive cases after obtaining the patients’ written consent (Supplementary Figure 3). Wherever possible, non-malignant tissues (*n* = 47) were taken each from a site at least 5 cm away from the tumor or collected from the patients attending the Endoscopy clinic in the Outpatient Department of Gastroenterology. Taken together, these 47 non-malignant esophageal tissues with histological evidence of normal epithelia constituted the normal group. After excision, tissues were immediately snap-frozen in liquid nitrogen and stored at –80°C in the Research Tissue Bank till further use; one part of the tissue was collected in 10% formalin and embedded in paraffin for histopathological and immunohistochemical analyses. Histologically confirmed esophageal normal epithelia, dysplasia, and ESCC as revealed by H&E staining were used for immunohistochemistry [28]. Only ESCC cases where the H&E section showed more than 80% of cancer cells were taken for biomarker analyses. The histologically confirmed normal tissues which did not show cancer cells or evidence of dysplasia were selected for biomarker analyses.

### Clinicopathological data

Patient demographic, clinical, and pathological data were recorded in a pre-designed Performa as described previously [28]. The information documented included clinical TNM staging (tumor, node, and metastasis based on the Union International Center le Cancer TNM classification of malignant tumors 2010), site of the lesion, histopathological differentiation, age, and gender. The treatment details were recorded and post-surgical treatment details were obtained from the clinical database. All patients received surgery as the primary treatment. Some patients received chemo therapy or radiation or combination of both as per the NCCN guidelines.

### Follow-up study

Eighty ESCC patients who underwent treatment from 2005–2010 could be investigated and evaluated in the esophageal cancer follow-up clinic at regular time intervals. Survival status of ESCC patients was verified and updated from the records of the Tumor Registry, Department of Gastrointestinal Surgery, AIIMS, as of June 2013. The median follow up for ESCC patients was 8.5 months (range 5–86 months). Disease-free survival time is defined as the time from completion of primary treatment till the patient showed any clinical and radiological evidence of local or regional disease, or distant metastasis at the time of the last follow-up of patients monitored in this study. Twenty eight patients who did not show recurrence were alive until the end of the follow-up period. Only disease-free survival was evaluated in this study, as the number of deaths due to disease progression did not allow a reliable statistical analysis.

### Immunohistochemistry

Paraffin-embedded sections (5 μm) of human esophageal histological normal (*n* = 47), dysplasia (*n* = 61) and ESCC (*n* = 80) were collected on gelatin-coated slides. The ESCC tissues analysed in this study had more than 80% tumor cells in H&E sections. In brief, the sections were deparaffinized in xylene, hydrated in gradient alcohol, and pre-treated in a microwave oven for 10 min at 800 W and 5 min at 480 W in Tris-EDTA (10 mM Tris, 1mM EDTA, pH = 9.0) for antigen retrieval. The sections were incubated with hydrogen peroxide (3% v/v) in methanol for 30 min to quench the endogenous peroxidise activity, followed by blocking with 1% bovine serum albumin (BSA) to preclude nonspecific binding. Thereafter, the slides were incubated with primary antibodies for 16 h at 4°C. The primary antibody was detected using the streptavidin-biotin complex with the Dako LSAB plus kit (Dako Cytomation, Glostrup, Denmark) and diaminobenzidine as the chromogen as described before [28]. In the negative control tissue sections, the primary antibody was replaced by isotype specific non-immune mouse IgG. A section from breast cancer tissue was used as a positive control in each batch of immunohistochemistry. Primary antibodies used in this study were as follows: E-cadherin (1:500; Santa Cruz, sc-8426), β-catenin (1:500; Santa Cruz, sc-7963), α-catenin (1:500; Santa Cruz, sc-7894), Dvl (1:500; Santa Cruz, sc-7397) and c-myc (1:200; Santa Cruz, sc-40).

### Evaluation of immunohistochemical staining

Each tissue section was evaluated for immunostaining using a semi-quantitative scoring system for both staining intensity and the percentage of positive epithelial cells [28]. For analysis of the expression of each protein, sections were scored as positive if epithelial cells showed immunopositivity in the nucleus/cytoplasm when observed independently by three of us (MRH, RS, SDG), who were blinded to the clinical outcome (the slides were coded and the scorers did not have prior knowledge of the local tumor burden, lymphonodular spread, and grading of the tissue samples). The tissue sections were scored based on the % of immunostained cells as: 0–10% = 0; 10–30% = 1; 31–50% = 2; 51–70% = 3 and ≥70% = 4. Sections were also scored semi-quantitatively on the basis of staining intensity as negative = 0; mild = 1; moderate = 2; intense =3. Finally, a total score was obtained by adding the score of percentage positivity and intensity. In cases where both nuclear and cytoplasmic immunoreactivity was observed, the nuclear and cytoplasmic staining was scored independently. The scoring by the three observers was discrepant in about 5% cases and a consensus on the final result was reached by re-evaluation of these slides and discussion.

### Statistical analyses

Descriptive analyses were performed on clinical and pathologic factors. Wilcoxon tests were used to assess relationships between biomarkers expression levels in subcellular compartments and lesion type (normal, dysplastic and ESCC). Univariate and multivariate Cox regression analyses were used to assess the prognostic value of biomarkers alone and when adjusted for clinical parameters: tumor stage, nodal status, and histology grade. A limit of 10 Events Per Variable (EPV) was used to avoid overfitting and yield stable models that perform relatively well on similar populations [29, 30]. Various Cox models were fitted, and assessed using a Likelihood Ratio Test (LRT) [31]. A signature score was derived using regression estimates of biomarkers that achieved the best model fit. Harrell’s c-statistic was used to assess discriminatory ability of all models [31]. Cox regression models were internally validated, and c-statistics were corrected for optimism using 9999 bootstrap sample sets [31]. Cox proportional hazards assumption was ensured using chi-squared test for goodness of fit on Schoenfeld residuals [32]. Recursive Partitioning (RP) analyses were done to identify terminal nodes that had significantly different survival curves [33]. Three years survival rates and the median survival time were used to describe survival curves of those nodes. The clinical relevance of biomarkers signature score in stratifying subjects into high and low risk group was assessed using sensitivity, specificity, Positive Predictive Value (PPV), Negative Predictive Value (NPV), area under the receiver operating characteristic curve (AUC), and accuracy. All statistical analyses were carried out using R version 3.01. Cox proportional hazard models were fitted using *rms* package in R [34]. RA trees were built using *party* R package [35].

## CONCLUSIONS

In conclusion, integrated analysis of expression of the panel of 4 proteins in ESCC patients has allowed us to validate the robustness of our biomarker panel in stratification of patients at high or low risk of disease recurrence. This risk classifier has the potential to identify the high risk patients for more rigorous personalized treatment and the low risk patients may be spared from the harmful side effects of toxic therapy as well reduce the burden on health care providers. The findings of our study set the foundations for external validation of the prognostic signature as a step forward in translation of this panel of protein markers for ESCC patients and establish their clinical relevance for larger worldwide application in future studies.

## SUPPLEMENTARY MATERIALS



## References

[1] Song Y , Li L , Ou Y , Gao Z , Li E , Li X , Zhang W , Wang J , Xu L , Zhou Y , Ma X , Liu L , Zhao Z , et al. Identification of genomic alterations in oesophageal squamous cell cancer. Nature. 2014; 509:91–95. 10.1038/nature13176. 24670651

[2] Blom RL , Lagarde SM , van Oudenaarde K , Klinkenbijl JH , Hulshof MC , van Laarhoven HW , Bergman JJ , Busch OR , van Berge Henegouwen MI . Survival after recurrent esophageal carcinoma has not improved over the past 18 years. Ann Surg Oncol. 2013; 20:2693–98. 10.1245/s10434-013-2936-3. 23549882

[3] Kim W , Kim M , Jho EH . Wnt/β-catenin signalling: from plasma membrane to nucleus. Biochem J. 2013; 450:9–21. 10.1042/BJ20121284. 23343194

[4] MacDonald BT , Tamai K , He X . Wnt/beta-catenin signaling: components, mechanisms, and diseases. Dev Cell. 2009; 17:9–26. 10.1016/j.devcel.2009.06.016. 19619488 PMC2861485

[5] Naganuma S , Whelan KA , Natsuizaka M , Kagawa S , Kinugasa H , Chang S , Subramanian H , Rhoades B , Ohashi S , Itoh H , Herlyn M , Diehl JA , Gimotty PA , et al. Notch receptor inhibition reveals the importance of cyclin D1 and Wnt signaling in invasive esophageal squamous cell carcinoma. Am J Cancer Res. 2012; 2:459–75. 22860235 PMC3410579

[6] Gan XQ , Wang JY , Xi Y , Wu ZL , Li YP , Li L . Nuclear Dvl, c-Jun, beta-catenin, and TCF form a complex leading to stabilization of beta-catenin-TCF interaction. J Cell Biol. 2008; 180:1087–100. 10.1083/jcb.200710050. 18347071 PMC2290839

[7] Hasan R , Sharma R , Saraya A , Chattopadhyay TK , DattaGupta S , Walfish PG , Chauhan SS , Ralhan R . Mitogen activated protein kinase kinase kinase 3 (MAP3K3/MEKK3) overexpression is an early event in esophageal tumorigenesis and is a predictor of poor disease prognosis. BMC Cancer. 2014; 14:2. 10.1186/1471-2407-14-2. 24383423 PMC3890584

[8] Hao XW , Zhu ST , He YL , Li P , Wang YJ , Zhang ST . Epigenetic inactivation of secreted frizzled-related protein 2 in esophageal squamous cell carcinoma. World J Gastroenterol. 2012; 18:532–40. 10.3748/wjg.v18.i6.532. 22363119 PMC3280398

[9] Kishino T , Niwa T , Yamashita S , Takahashi T , Nakazato H , Nakajima T , Igaki H , Tachimori Y , Suzuki Y , Ushijima T . Integrated analysis of DNA methylation and mutations in esophageal squamous cell carcinoma. Mol Carcinog. 2016; 55:2077–88. 10.1002/mc.22452. 26756304

[10] Ge XS , Ma HJ , Zheng XH , Ruan HL , Liao XY , Xue WQ , Chen YB , Zhang Y , Jia WH . HOTAIR, a prognostic factor in esophageal squamous cell carcinoma, inhibits WIF-1 expression and activates Wnt pathway. Cancer Sci. 2013; 104:1675–82. 10.1111/cas.12296. 24118380 PMC7653522

[11] Moghbeli M , Abbaszadegan MR , Farshchian M , Montazer M , Raeisossadati R , Abdollahi A , Forghanifard MM . Association of PYGO2 and EGFR in esophageal squamous cell carcinoma. Med Oncol. 2013; 30:516. 10.1007/s12032-013-0516-9. 23456637

[12] Ninomiya I , Endo Y , Fushida S , Sasagawa T , Miyashita T , Fujimura T , Nishimura G , Tani T , Hashimoto T , Yagi M , Shimizu K , Ohta T , Yonemura Y , et al. Alteration of beta-catenin expression in esophageal squamous-cell carcinoma. Int J Cancer. 2000; 85:757–61. 10.1002/(sici)1097-0215(20000315)85:6<757::aid-ijc3>3.0.co;2-o. 10709091

[13] Salahshor S , Naidoo R , Serra S , Shih W , Tsao MS , Chetty R , Woodgett JR . Frequent accumulation of nuclear E-cadherin and alterations in the Wnt signaling pathway in esophageal squamous cell carcinomas. Mod Pathol. 2008; 21:271–81. 10.1038/modpathol.3800990. 18084253

[14] Mizutani K , Miyamoto S , Nagahata T , Konishi N , Emi M , Onda M . Upregulation and overexpression of DVL1, the human counterpart of the Drosophila dishevelled gene, in prostate cancer. Tumori. 2005; 91:546–51. 16457155 10.1177/030089160509100616

[15] Uematsu K , He B , You L , Xu Z , McCormick F , Jablons DM . Activation of the Wnt pathway in non small cell lung cancer: evidence of dishevelled overexpression. Oncogene. 2003; 22:7218–21. 10.1038/sj.onc.1206817. 14562050

[16] Wei Q , Zhao Y , Yang ZQ , Dong QZ , Dong XJ , Han Y , Zhao C , Wang EH . Dishevelled family proteins are expressed in non-small cell lung cancer and function differentially on tumor progression. Lung Cancer. 2008; 62:181–92. 10.1016/j.lungcan.2008.06.018. 18692936

[17] Zhao Y , Yang ZQ , Wang Y , Miao Y , Liu Y , Dai SD , Han Y , Wang EH . Dishevelled-1 and dishevelled-3 affect cell invasion mainly through canonical and noncanonical Wnt pathway, respectively, and associate with poor prognosis in nonsmall cell lung cancer. Mol Carcinog. 2010; 49:760–70. 10.1002/mc.20651. 20572159

[18] Wang W , Li X , Lee M , Jun S , Aziz KE , Feng L , Tran MK , Li N , McCrea PD , Park JI , Chen J . FOXKs promote Wnt/β-catenin signaling by translocating DVL into the nucleus. Dev Cell. 2015; 32:707–18. 10.1016/j.devcel.2015.01.031. 25805136 PMC4374128

[19] Hawksworth D , Ravindranath L , Chen Y , Furusato B , Sesterhenn IA , McLeod DG , Srivastava S , Petrovics G . Overexpression of C-MYC oncogene in prostate cancer predicts biochemical recurrence. Prostate Cancer Prostatic Dis. 2010; 13:311–15. 10.1038/pcan.2010.31. 20820186

[20] Long X , Hu S , Cao P , Liu Z , Zhen H , Cui Y . [The expression of oncogene c-myc and its role on human laryngeal cancer]. Lin Chung Er Bi Yan Hou Tou Jing Wai Ke Za Zhi. 2009; 23:1127–29. 20359089

[21] Zhang JX , Tong ZT , Yang L , Wang F , Chai HP , Zhang F , Xie MR , Zhang AL , Wu LM , Hong H , Yin L , Wang H , Wang HY , Zhao Y . PITX2: a promising predictive biomarker of patients’ prognosis and chemoradioresistance in esophageal squamous cell carcinoma. Int J Cancer. 2013; 132:2567–77. 10.1002/ijc.27930. 23132660

[22] Chen J , Yang H , Wen J , Luo K , Liu Q , Huang Y , Zheng Y , Tan Z , Huang Q , Fu J . NHE9 induces chemoradiotherapy resistance in esophageal squamous cell carcinoma by upregulating the Src/Akt/β-catenin pathway and Bcl-2 expression. Oncotarget. 2015; 6:12405–20. 10.18632/oncotarget.3618. 25915159 PMC4494947

[23] Qian X , Tan C , Wang F , Yang B , Ge Y , Guan Z , Cai J . Esophageal cancer stem cells and implications for future therapeutics. Onco Targets Ther. 2016; 9:2247–54. 10.2147/OTT.S103179. 27143920 PMC4846051

[24] Wu J , Ru NY , Zhang Y , Li Y , Wei D , Ren Z , Huang XF , Chen ZN , Bian H . HAb18G/CD147 promotes epithelial-mesenchymal transition through TGF-β signaling and is transcriptionally regulated by Slug. Oncogene. 2011; 30:4410–27. 10.1038/onc.2011.149. 21532623

[25] Natsuizaka M , Ohashi S , Wong GS , Ahmadi A , Kalman RA , Budo D , Klein-Szanto AJ , Herlyn M , Diehl JA , Nakagawa H . Insulin-like growth factor-binding protein-3 promotes transforming growth factor-{beta}1-mediated epithelial-to-mesenchymal transition and motility in transformed human esophageal cells. Carcinogenesis. 2010; 31:1344–53. 10.1093/carcin/bgq108. 20513670 PMC2915630

[26] Hong S , Lee HJ , Kim SJ , Hahm KB . Connection between inflammation and carcinogenesis in gastrointestinal tract: focus on TGF-beta signaling. World J Gastroenterol. 2010; 16:2080–93. 10.3748/wjg.v16.i17.2080. 20440848 PMC2864833

[27] Mendelson J , Song S , Li Y , Maru DM , Mishra B , Davila M , Hofstetter WL , Mishra L . Dysfunctional transforming growth factor-β signaling with constitutively active Notch signaling in Barrett’s esophageal adenocarcinoma. Cancer. 2011; 117:3691–702. 10.1002/cncr.25861. 21305538 PMC3236645

[28] Hasan MR , Sharma R , Saraya A , Chattopadhyay TK , DattaGupta S , Walfish PG , Chauhan SS , Ralhan R . Slug is a predictor of poor prognosis in esophageal squamous cell carcinoma patients. PLoS One. 2013; 8:e82846. 10.1371/journal.pone.0082846. 24367561 PMC3867395

[29] Peduzzi P , Concato J , Kemper E , Holford TR , Feinstein AR . A simulation study of the number of events per variable in logistic regression analysis. J Clin Epidemiol. 1996; 49:1373–79. 10.1016/s0895-4356(96)00236-3. 8970487

[30] Burnham KP , Anderson DR . Model Selection and Inference: A Practical Information-theoretic Approach: with 21 Illustrations: Springer-Verlag GmbH. 1998.

[31] Harrell FE . Regression Modeling Strategies: With Applications to Linear Models, Logistic Regression, and Survival Analysis: Springer. 2001.

[32] Grambsch PM , Therneau TM . Proportional hazards tests and diagnostics based on weighted residuals. Biometrika. 1994; 81:515–26. 10.2147/OTT.S103179.

[33] Strobl C , Malley J , Tutz G . An introduction to recursive partitioning: rationale, application, and characteristics of classification and regression trees, bagging, and random forests. Psychol Methods. 2009; 14:323–48. 10.1037/a0016973. 19968396 PMC2927982

[34] Harrell FE Jr . rms: Regression Modeling Strategies. 2013.

[35] Hothorn T , Hornik K , Strobl C , Zeileis A . party: A Laboratory for Recursive Partytioning. 2013.

