# Supplementary material for: Prediction of recurrence free survival for esophageal cancer patients using a protein signature based risk model
Source: Oncotarget. 2022 Sep 14;13:1020–32. doi: 10.18632/oncotarget.10656 (PMC9477219; doi:10.18632/oncotarget.10656)
Supplement: Supplementary file 1 [file oncotarget-13-10656-s001.pdf]

Prediction of recurrence free survival for esophageal cancer patients using a protein signature based risk model

SUPPLEMENTARY MATERIALS

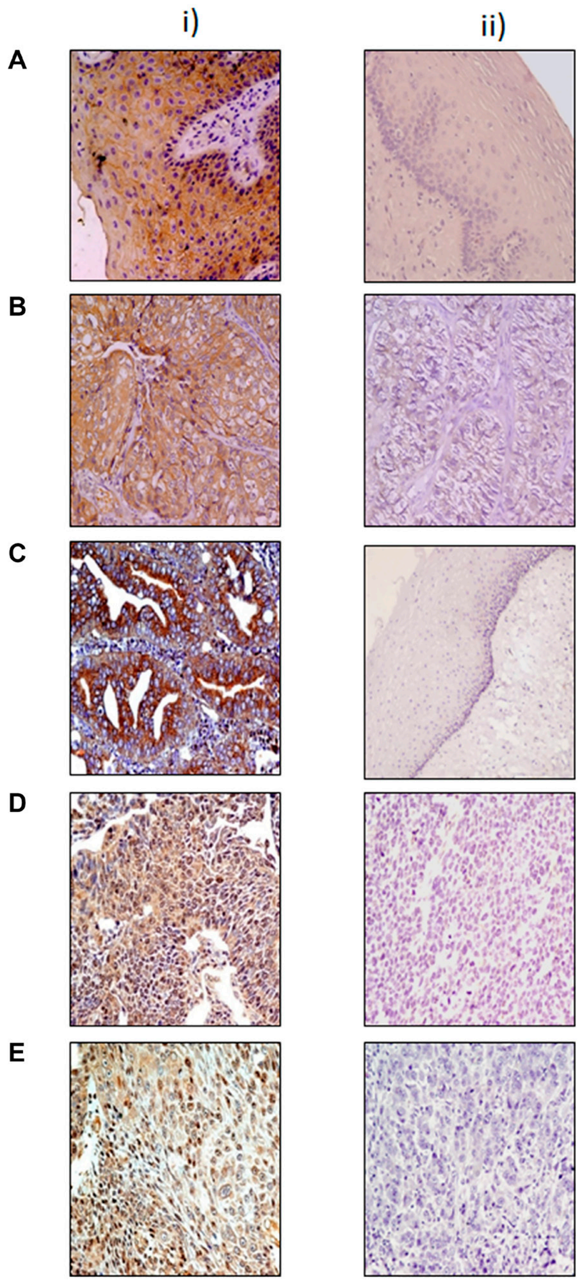

Supplementary Figure 1: Photomicrograph depicting positive and negative controls for immunostaining for Wnt proteins. Panel (i) shows immunostaining of: (**A**) β-catenin in normal oral tissue used as a positive control showing strong membranous immunoreactivity. (**B**) E-cadherin in breast carcinoma tissue used as positive control showing strong membranous and cytoplasmic immunostaining. (**C**) α-catenin in gastric carcinoma tissue used as positive control showing strong membranous and cytoplasmic immunoreactivity. (**D**) Dishevelled (Dvl) in esophageal carcinoma tissue used as positive control showing strong cytoplasmic immunoreactivity. (**E**) C-myc in esophageal carcinoma tissue used as positive control showing strong nuclear immunoreactivity. Panel (ii) shows the respective negative controls for each of the above protein where the primary antibody was replaced with IgG showing no detectable protein expression. (Original magnification panels 1 and 2 A–E ×200).

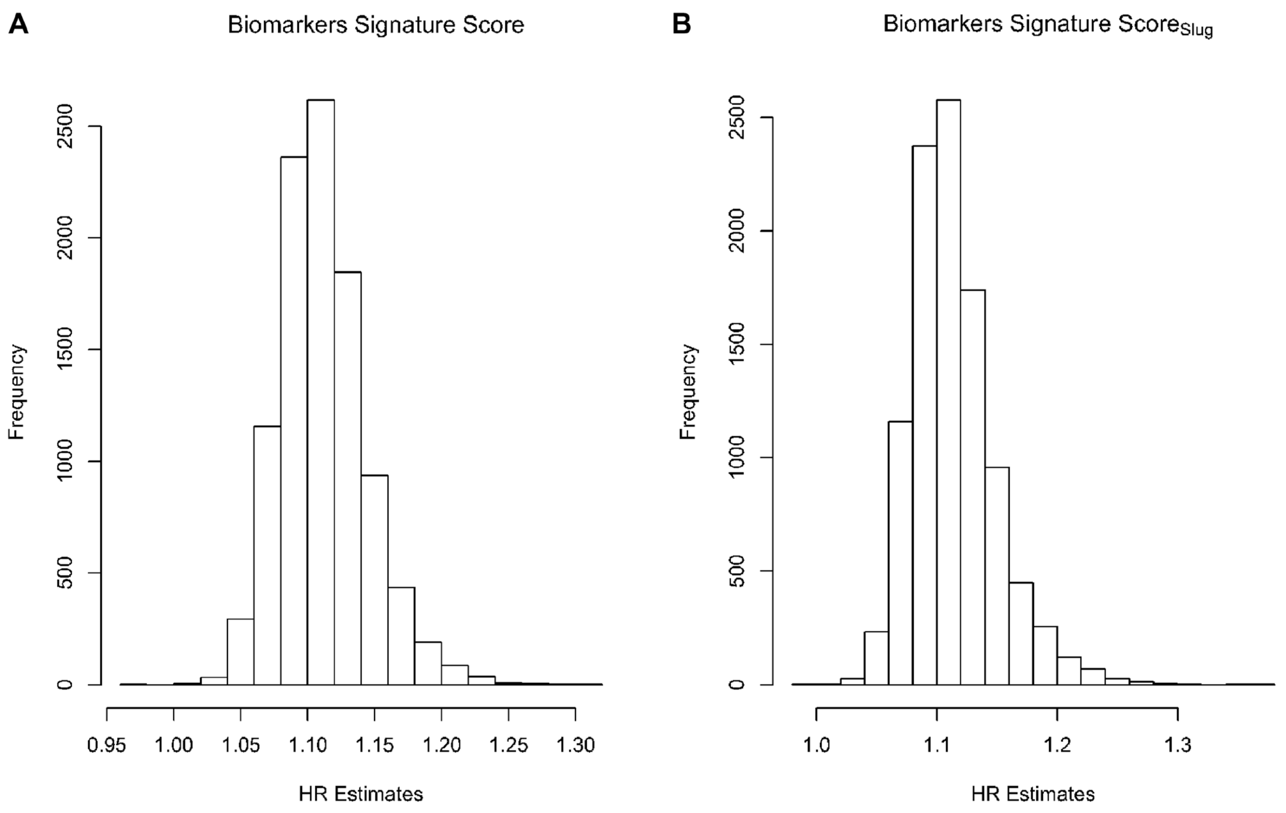

Supplementary Figure 2: Sample distribution of HR estimates of biomarker signature score, biomarker signature score$_{slug}$ and biomarker depicted score. Both distributions are approximately normal.

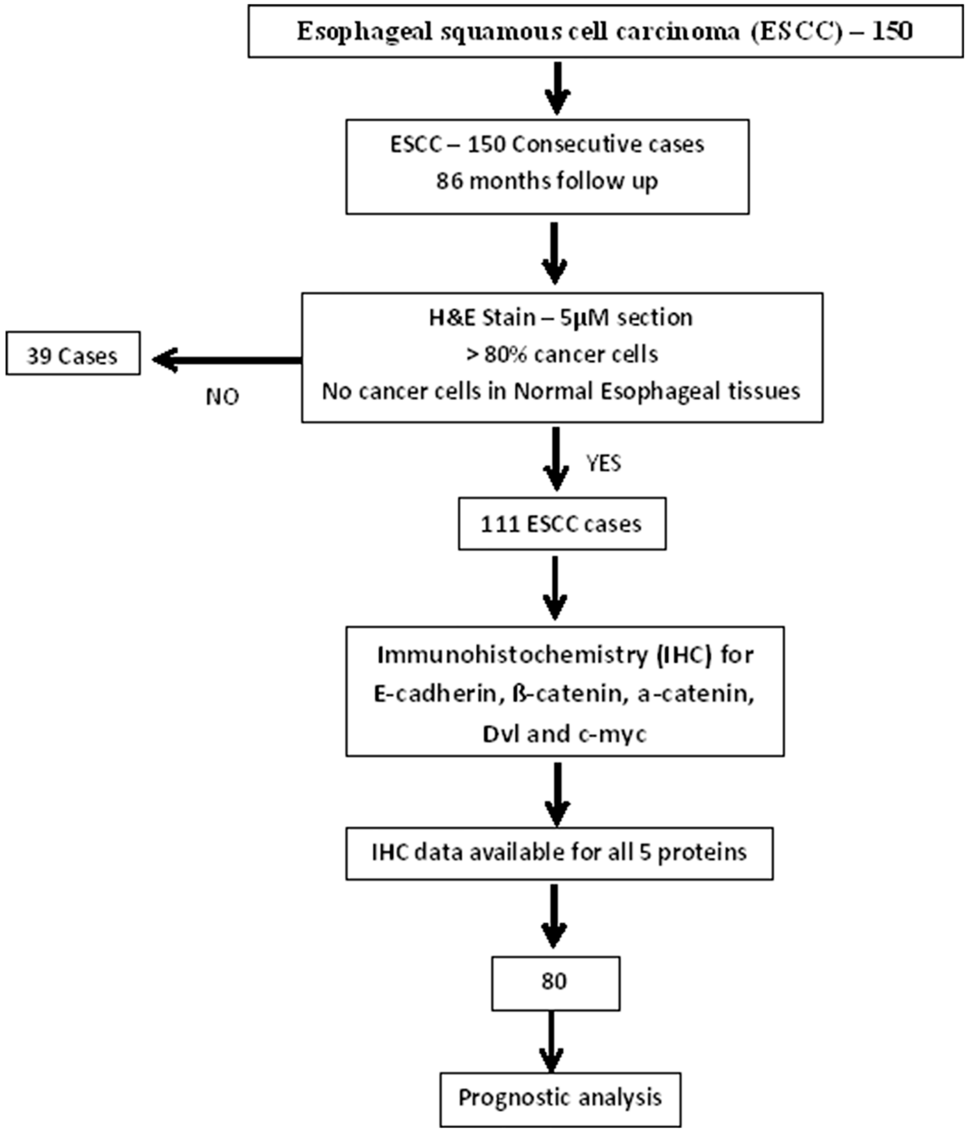

Supplementary Figure 3: Diagram showing study design, case selection and Immunohistochemical analysis.

Supplementary Table 1: Potential Cox regression models

Predictors	c-statistic	LRT p	AIC
membrane β-catenin + cytoplasmic DVL + nuclear DVL	0.62	0.09	294.73
membrane β-catenin + nuclear c-MyC + nuclear DVL	0.64	0.06	294.09
cytoplasmic β-catenin + cytoplasmic DVL + nuclear DVL	0.64	0.02	292.40
membrane α-catenin + cytoplasmic DVL + nuclear DVL	0.64	0.03	293.82
cytoplasmic β-catenin + nuclear c-MyC + nuclear DVL	0.67	0.01	291.84
cytoplasmic β-catenin + membrane β-catenin + nuclear c-MyC + nuclear DVL	0.68	0.02	293.28

Supplementary Table 2: Bootstrap of univariate & multivariable analyses

Predictors	Univariate analyses		Multivariable analyses	
	HR [95% CI]	*p*	HR [95% CI]	*p*
Cytoplasmic β-catenin	1.16 [1.04, 1.30]	0.01	1.19 [1.06, 1.36]	0.01
Membrane α-catenin	0.86 [0.7, 0.99]	0.22	0.83 [0.64, 1.00]	0.25
Nuclear DVL	1.13 [1.04, 1.25]	0.01	1.12 [1.02, 1.28]	0.05
Nuclear c-MyC	1.09 [0.96, 1.24]	0.24	1.14 [0.98, 1.38]	0.17
Biomarker Score	1.11 [1.06, 1.19]	< 0.001	1.12 [1.06, 1.22]	0.004
Nuclear Slug	1.22 [1.1, 1.38]	< 0.001	1.22 [1.08, 1.41]	0.006
Score _ Slug	1.11 [1.06, 1.20]	0.001	1.11 [1.05, 1.23]	0.01
Tumor stage	2.97 [1.03, 1394.84]	0.59		
Nodal Status	1.74 [0.85, 4.11]	0.15		
Histological grade	1.06 [0.55, 2.03]	0.84		
Radiation therapy	0.73 [0.38, 1.42]	0.36		
Chemotherapy	0.92 [0.05, 7.68]	0.92		

Validations were based on 9999 bootstrap random samples.

Supplementary Table 3: Bootstrap validations of signature scores' clinical relevance

Clinical value	Biomarker signature score	Biomarker signature score$_{slug}$
	**1 Year**	
	**High vs. Low Risk Groups**	**High vs. Low Risk Groups**
Sensitivity	0.50	0.77
Specificity	0.84	0.76
PPV	0.79	0.80
NPV	0.59	0.73
AUC	0.67	0.76
	**3 Years**	
	**High vs. Low Risk Groups**	**High vs. Low Risk Groups**
Sensitivity	0.45	0.67
Specificity	1.00	0.86
PPV	1.00	0.97
NPV	0.23	0.30
AUC	0.73	0.76

Score 1 refers to cytoplasmic β-catenin, nuclear c-MyC, nuclear DVL and membrane α-catenin signature score (cut-off value = 8.28). Score 2 refers Score with nuclear Slug instead of membrane (cut-off value = 6.50).
